# Determination of an optimal response cut-off able to predict progression-free survival in patients with well-differentiated advanced pancreatic neuroendocrine tumours treated with sunitinib: an alternative to the current RECIST-defined response

**DOI:** 10.1038/bjc.2017.402

**Published:** 2017-11-21

**Authors:** Angela Lamarca, Jorge Barriuso, Matthew Kulke, Ivan Borbath, Heinz-Josef Lenz, Jean Luc Raoul, Neal J Meropol, Catherine Lombard-Bohas, James Posey, Sandrine Faivre, Eric Raymond, Juan W Valle

**Affiliations:** 1 Department of Medical Oncology, The Christie NHS Foundation Trust (ENETS Centre of Excellence), Manchester M20 4BX, UK; 2 Faculty of Medical, Biological and Human Sciences, University of Manchester, Manchester M13 9GB, UK; 3 Department of Medical Oncology, Dana-Farber Cancer Institute, Boston, MA 02215, USA; 4 Department of Gastroenterology, Cliniques Universitaires Saint-Luc, Bruxelles 1200, Belgium; 5 University of Southern California/Norris Comprehensive Cancer Center, Los Angeles, CA 90033, USA; 6 Paoli-Calmettes Institute, Marseille 13009, France; 7 Case Comprehensive Cancer Center, Case Western Reserve University, Cleveland, OH 44106, USA; 8 Flatiron Health, New York, NY 10010, USA; 9 Department of Medical Oncology, Hospices Civils de Lyon Edouard Herriot Hospital, University of Lyon, Lyon 69002, France; 10 Department of Medical Oncology, Sidney Kimmel Cancer Center, Thomas Jefferson University, Philadelphia, PA 19107, USA; 11 Department of Medical Oncology, Beaujon University Hospital, Paris 92110, France; 12 Department of Medical Oncology, Groupe Hospitalier Paris Saint-Joseph, Paris 75014, France; 13 Institute of Cancer Sciences, University of Manchester, Manchester M204BX, UK

**Keywords:** radiological response, RECIST, sunitinib, progression-free survival, neuroendocrine tumour, pancreas

## Abstract

**Background:**

Sunitinib prolongs progression-free survival (PFS) in patients with advanced pancreatic neuroendocrine tumours (pNET). Response Evaluation Criteria in Solid Tumors (RECIST)-defined partial responses (PR; classically defined as ⩾30% size decrease from baseline) are infrequent.

**Methods:**

Individual data of pNET patients from the phase II [NCT00056693] and pivotal phase III [NCT00428597] trials of sunitinib were analysed in this investigator-initiated, *post hoc* study. The primary objective was to determine the optimal RECIST (v.1.0) response cut-off value to identify patients who were progression-free at 11 months (median PFS in phase III trial); and the most informative time-point (highest area under the curve (AUC) by receiver operating characteristic (ROC) analysis and logistic regression) for prediction of benefit (PFS) from sunitinib.

**Results:**

Data for 237 patients (85 placebo; 152 sunitinib (*n*=66.50 mg ‘4-weeks on/2-weeks off’ schedule; *n*=86 ‘37.5 mg continuous daily dosing (CDD)’)) and 788 scans were analysed. The median PFS for sunitinib and placebo were 9.3 months (95% CI 7.6–12.2) and 5.4 months (95% CI 3.5–6.01), respectively (hazard ratio (HR) 0.43 (95% CI 0.29–0.62); *P*<0.001). A PR was seen in 19 patients (13%) on sunitinib; the median change in the sum of the lesions (*vs* baseline) was −12.8% (range −100 to +36.4). Month 7 was the most informative time-point (AUC 0.78 (95% CI 0.66–0.9); odds ratio 1.05 (95% CI 1.01–1.08), *P*=0.002). Reduction of 10% (*vs* baseline) achieved the highest sensitivity (50%) and specificity (82%), amongst cut-offs tested. A 10% reduction in marker lesions was associated with improved PFS in the whole sunitinib population (HR 0.55 (95 CI 0.3–0.9); *P*=0.04); mostly in patients on sunitinib CDD (HR 0.33 (95% CI 0.2–0.7); *P*=0.005). A 10% reduction in marker lesions (*P*=0.034) and sunitinib treatment (*P*=0.012) independently impacted on PFS (multivariable analysis).

**Conclusions:**

A 10% reduction within marker lesions identifies pNET patients benefiting from sunitinib treatment with implications for maintenance of dose intensity and future trial design.

Assessment of the change in tumour burden is an important feature of the clinical evaluation of anticancer therapies. Response Evaluation Criteria in Solid Tumours (RECIST) were reviewed in 2000 (Therasse *et al*, 2000) and 2009 (Eisenhauer *et al*, 2009) and are widely employed in clinical trial and daily practice settings for assessing response to treatment. With the development of new targeted agents, the ability of RECIST to identify patients deriving benefit from treatment has been questioned. The main reason is because of the low rate of objective responses seen with targeted agents, which does not seem to be a meaningful surrogate of biological effect (where measures of improvement in progression-free, morphological changes or overall survival are more appropriate) (Faivre *et al*, 2012).

Pancreatic neuroendocrine tumours (pNETs) are rare tumours, for which targeted agents, such as sunitinib (Raymond *et al*, 2011) or everolimus (Yao *et al*, 2011), have shown benefit by significantly improving progression-free survival (PFS) in phase III trials compared to placebo controls. Data supporting the use of sunitinib in particular include a single-arm phase II study, using 50 mg per day in the classical ‘4 weeks on/2 weeks off’ schedule (Kulke *et al*, 2008); and a randomised phase III study using 37.5 mg continuous daily dosing (CDD) schedule (Raymond *et al*, 2011) achieving response rates (by RECIST v1.0 (Therasse *et al*, 2000)) of 16.7% and 9.3%, respectively. The pivotal phase III clinical trial (Raymond *et al*, 2011), reported by Raymond *et al* in 2011, randomised 171 patients diagnosed with advanced, progressive, well-differentiated pNET to receive either CDD sunitinib (37.5 mg daily) or placebo. The primary end-point of the study was median PFS; this clinical trial identified significant longer PFS in patients receiving sunitinib (11.4 versus 5.5 months; Hazard Ratio (HR) 0.42 (95% confidence interval (CI), 0.26–0.66; *P*<0.001); there were eight objective partial responses (9.3%) with sunitinib (versus none in the placebo group). Diarrhoea, nausea, vomiting, fatigue, hand-foot skin reaction and hypertension were the most common side effects.

Due to the low rate of partial response achieved with targeted agents when the classical 30% tumour shrinkage cut-off is employed (per RECIST v1.0 (Therasse *et al*, 2000) and v.1.1 (Eisenhauer *et al*, 2009)), and the questioned capacity of such definition to identify patients benefiting from such treatment (in terms of PFS), alternative cut-offs have been explored. As an example, a modification of RECIST has been proposed for patients with renal cell carcinoma (Thiam *et al*, 2010; Krajewski *et al*, 2011, 2014); (Krajewski *et al* 2011, 2014) validated a different cut-off for RECIST, able to identify more accurately those patients with benefit (in terms of overall survival) from anti-angiogenic agents (including sunitinib). Unfortunately, this has not been tested in pNET as yet and therefore, partial response as per the current definition (30% reduction) remains of limited value for patients’ management.

The aim of this analysis was to identify an alternative cut-off for definition of partial response able to predict clinical benefit from treatment with sunitinib in patients diagnosed with pNET.

## Materials and methods

### Study design

Patients diagnosed with well- and moderately differentiated pNETs who received treatment with sunitinib/placebo as part of the phase III (NCT00428597 (Raymond *et al*, 2011)) and phase II (NCT00428597 (Kulke *et al*, 2008)) clinical trials were eligible for this investigator-initiated *post hoc* analysis. Patients with other diagnoses, such as small bowel primary NET (included in the phase II (NCT00428597 (Kulke *et al*, 2008)) clinical trial) were excluded. No other exclusion criteria were applied. This study was approved by Pfizer, who facilitated access to anonymised individual-patient data (all patients had previously provided informed consent to be involved in the above-mentioned studies); all data employed in this study have previously been collected by Pfizer and quality-assured for registration purposes. Study was sponsored by The University of Manchester. Updated outcome data (July 2014) including open-label extension phase was included into this analysis.

### Study population

All clinical data, including demographics, treatment characteristics and radiological response assessment (as per investigator assessment), together with progression-free and overall survival collected within the above-mentioned studies were retrieved. Best response was classified as complete response, partial response, stable disease or progressive disease based on previously reported assessment by investigators involved in the phase II and phase III studies (RECIST v1.0 was employed) (Therasse *et al*, 2000). Changes within sum of tumour diameter compared to baseline/nadir in each one of the radiological assessment perfumed for each individual patient was calculated following RECIST v1.0 for this post hoc analysis (Therasse *et al*, 2000); should the measurement of one of the marker lesion be unavailable for a specific radiological assessment, such radiological assessment was considered not-evaluable.

### Study objectives

The primary objective of this *post hoc* analysis was to determine an alternative cut-off to the currently employed reduction of 30% of sum of marker lesions diameter in order to define partial response. We aimed to find the most informative RECIST response cut-off value (identified as the percentage cut-off value with maximum sensitivity and specificity in the ROC analysis evaluating patients as progression-free at 11 months); this time-point was pre-defined as the median PFS observed in the pivotal phase III study was 11.4 months.

Secondary objectives included (a) identification of the most informative assessment time-point (identified by the highest AUC) for prediction of clinical benefit from sunitinib (defined as progression-free at 11 months), (b) analysis of the impact of the new RECIST cut-off in prediction of PFS in patients treated with sunitinib/placebo, and (c) identification of the time-point with higher rate of best-response.

### Statistical analysis

No formal sample size calculation was performed. All eligible patients were included in the analysis. Due to limited sample size, it was not feasible to divide the sunitinib-treated patients into training and validation cohorts; therefore, all sunitinib-treated patients were analysed together.

Receiver operating characteristic (ROC) analysis was used to identify the most informative scan time-point (month 1, month 2, month 3, month 5, month 7, month 9 and month 11 and all radiological assessment performed thereafter) by plotting RECIST evaluation against PFS dichotomised by progression-free status at 11 months (yes *vs* no). This was facilitated by the fact that both trials (NCT00056693 and NCT00428597) had the radiological reassessment performed at these same time-points. The most suitable time-point was selected by comparing the area under the curve (AUC) from the ROC curves for each time-point and by performing logistic regression analysis. Those time-points with highest AUC (AUC of ⩾0.7 were pre-defined as being ‘of interest’) which also achieved statistically significant prediction by logistic regression (two sided *P*-value<0.05) were compared by ROC curve comparison analysis for selection of the most informative radiological assessment time-point. From this selected time-point, the performance of 30% reduction and other alternative cut-offs (20, 15 and 10%) were tested for prediction of progression-free at 11 months: guided by the highest sensitivity and specificity an alternative cut-off for new definition of partial response was identified. This alternative cut-off was employed to the achieved best-response for survival analysis.

Log-rank test, Kaplan-Meier method and Cox-regression (univariate) were used to assess the impact of both the standard 30% cut-off and our newly-defined cut-off on PFS. PFS was defined as the time from first-sunitinib dose (applicable for patients included in the phase II) or randomisation (applicable for patients included in the phase III) to the first evidence of progression or death from any cause. Survival analysis was replicated in patients treated with placebo with the intention of clarifying whether radiological response was a prognostic or a predictive factor. A multivariable analysis (Cox regression) adjusted for other known prognostic factors was performed.

## Results

A total of 237 patients and 788 scans were included in this *post hoc* analysis. Data for a total of 152 sunitinib-treated patients (66 from the phase II study and 86 from the phase III study) and 85 patients treated with placebo was received (Supplementary Figure 1).

### Patient characteristics, response to treatment and outcomes

The median age of the whole population was 56 years (range 25–84years), most patients were of Eastern Cooperative Oncology Group performance status (ECOG-PS) 0 (54%) or 1 (45%). The dose of sunitinib/placebo was 37.5 mg daily for all patients included in the phase III study; patients included into the phase II received 50 mg daily of sunitinib on a ‘4 weeks on, 2 weeks off’ schedule. Table 1 summarises baseline characteristics of the patients included in our analysis.

**Figure 1:**
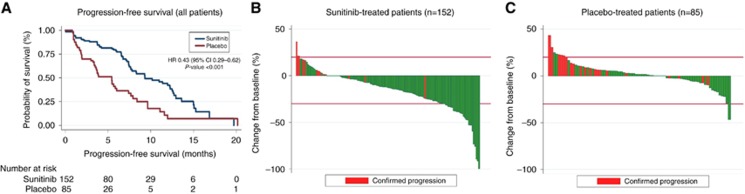
**Outcome of patients treated with sunitib/placebo.** Progression-free survival in patients with sunitinib and placebo (**A**); Waterfall-plot showing best changes in sum of marker lesions in patients treated with sunitinib (**B**) and placebo (**C**). HR, hazard ratio; 95% CI, 95% confidence interval; *n*, number of patients.

Patient outcomes are summarised in Table 1. The median follow-up was 16.5 months for the whole population (12, 32.5 and 23.2 months for patients treated with sunitinib in the phase II, phase III and placebo arm patients, respectively; Supplementary Table 1). Patients treated with sunitinib (all patients) and placebo achieved a median PFS of 9.3 months (95% CI 7.6–12.2) and 5.4 months (95% CI 3.5–6.01), respectively; hazard ratio (HR) 0.43 (95% CI 0.29–0.62), *P*-value <0.001 (Figure 1A). Median PFS was 9.3 (95% CI 7.1–11.9) and 12.6 (95% CI 7.4–16.8) for sunitinib-treated patients in the phase II and phase III, respectively (Supplementary Table 1).

Out of the whole population, 8% of patients were classified by the local investigator to have achieved a partial response: 13% of patients treated with sunitinib and 0% of patients in the placebo treatment. As per calculations performed in this *post hoc* analysis, the median time to best-response was 3 months in the sunitinib arm and 83.7% of patients achieved the best response by month 7 of treatment (Table 1 and Supplementary Table 2). Median best change in the sum of marker lesions as per calculations performed in this *post hoc* analysis was −12.8% and +1.7% for patients with sunitinib and placebo, respectively (Table 1, Figure 1B and C).

### Identification of the most informative time-point

Radiological assessment performed at month 5, month 7 and at the time of ‘best-response’ were the three time-points achieving both an AUC higher than 0.7 and statistically significant prediction of being progression-free at 11 months (logistic regression) (Table 2). Therefore, these three time-points were compared by ROC curve evaluation: although differences between the three time-points were not statistically significant (*P*-value 0.3733 (Supplementary Figure 2), month 7 maintained the highest AUC (0.75 (95% CI 0.63–0.86) and was therefore selected as the most informative time-point. The observed median time on treatment in the sunitinib group was 6.4 months (Table 1) supported this approach.

### Alternative cut-off for new definition of ‘partial response’

Fifty-four scans were available at month 7 and were used for the identification of the alternative response cut-off. Performance of 30% reduction in sum of marker lesions and other alternative cut-offs (20, 15 and 10%) were tested for prediction of being progression-free at 11 months (Table 3). A RECIST reduction of 10% was the most informative cut-off with highest rate of correctly classified patients (66.7%), maintaining an admissible specificity (82%). Since month 5 achieved a similar AUC to month 7 at the identification of the most informative time-point (Table 2 and Supplementary Material 4), alternative cut-offs were tested in this time-point, to confirm the consistency of our alternative cut-off (Supplementary Material 4). In addition, when this 10% cut-off was applied to the best-response scans, it significantly increased the number of patients classified as ‘partial response’ (59%), compared to the more restrictive 30% cut-off, which classified ‘partial responses’ in 20% of patients.

### Survival analysis

**Figure 2:**
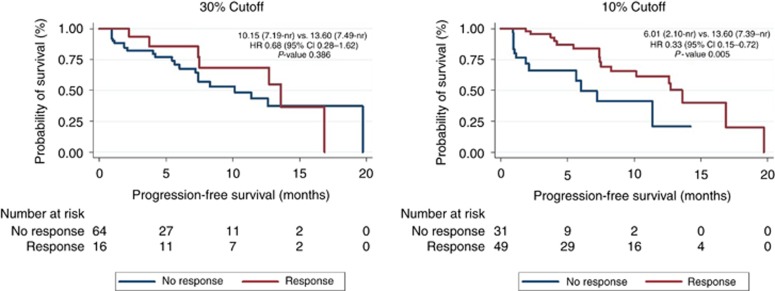
**Progression free-survival in patients treated with 37.5 mg continuously of sunitinib (Phase III clinical trial), both cut-offs (classical 30% and our proposed alternative 10%) are tested.** CI=confidence interval; HR=hazard ratio; 95% nr=not-reached.

Survival analysis was employed to compare the impact on PFS of achieving a partial response according to the classical 30% cut-off or our proposed alternative (10% reduction). Univariate analysis confirmed that the 10% reduction in sum of marker lesions cut-off impacted PFS for sunitinib-treated patients (HR 0.55 (95% CI 0.31–0.97); *P*-value 0.04), while the 30% cut-off did not (*P*-value 0.198) (Table 4). The benefit was even more marked when the analysis was limited to the phase III sunitinib-treated patients (37.5 mg CDD) were analysed (HR 0.33 (95% CI 0.15–0.72); *P*-value 0.005; median PFS for patients achieving 10% reduction was 13.60 months (95% CI 7.39-not reached) and it was 6.01 months 95% CI 2.10–not reached) for those who did not (Figure 2)). Similar results were achieved in the placebo-treated patients. Results were not reproduced in the phase II study (*P*-value 0.980). See Table 4 for full details.

Multivariable analysis confirmed that both treatment with sunitinib and achieving a 10% of reduction in tumour diameter were independent prognostic factors for longer PFS (Table 4); achieving a response of 30% was not an independent prognostic factor in the multivariable analysis (see Supplementary Table 3) for full detail regarding univariate and multivariable analysis).

## Discussion

There is a need for optimising our currently-available tools for radiological assessment of response in patients with NETs. This could be done by (1) maximising the information provided by current standard size-based criteria (such as RECIST), (2) incorporation of morphological assessment (e.g., Choi criteria (Faivre *et al*, 2012)) or by (3) incorporating metabolic techniques (nuclear medicine (Sundin and Rockall, 2012)) into response assessment. This study focused on the first approach.

This *post hoc* analysis is the first study to identify an alternative tumour shrinkage cut-off for definition of partial response in sunitinib-treated pNET patients. While the classical cut-off of 30% of tumour diameter reduction was shown to be too restrictive and not impacting PFS in the multivariable analysis, our proposed alternative of 10% reduction did impact PFS, even when adjusted to other prognostic factors.

Best-response to treatment was achieved early-on in the treatment with sunitinib: at a median of 3 months to best-response, with 83.9% of patients achieving the best-response during the first 7 months of treatment. Therefore, this alternative definition of objective partial response can be used as an early marker of benefit from treatment and could impact patients’ management.

Our results support that a reduction of 10% may be used as an accurate surrogate for PFS. Earlier studies have shown the importance of maintenance of dose intensity in sunitinib-treated patients in renal cell carcinoma (RCC) (Houk *et al*, 2010). We would therefore suggest that dose reduction rather than dose interruption is considered in the event of treatment-related toxicity for those patients who have achieved our suggested 10% reduction on the size of targeted lesions, in accordance with the prescribing information. Moreover, since, as mentioned above, the best-response was achieved early-on following initiation of treatment, we also argue that should patients not achieve a 10% of tumour shrinkage after 7 months of treatment, the dose of sunitinib could be increased to 50 mg daily (if well tolerated) as suggested in the sunitinib SPC (EMA, 2016) and as detailed in the phase III clinical trial protocol which stated that ‘in patients without an objective tumour response who had grade 1 or lower non-haematologic or grade 2 or lower haematologic treatment-related adverse events during the first 8 weeks, the dose could be increased to 50 mg per day’ (Raymond *et al*, 2011). Although dose escalation of sunitinib has been explored in RCC and gastrointestinal stromal tumour (GIST) (Patel, 2012; Ornstein *et al*, 2016; Shi *et al*, 2016), experience of doing so in pNET is limited; only 10% of patients treated with sunitinib in the phase III pivotal clinical trial had dose increased to 50 mg daily, and its impact is unclear (Raymond *et al*, 2011).

Alternative response cut-offs have also been explored in the past in other malignancies such as RCC, in which targeted therapies (including sunitinib) are a cornerstone of systemic management (Thiam *et al*, 2010, Krajewski *et al*, 2011, 2014). (Krajewski *et al* 2011, 2014) validated a different cut-off for RECIST criteria, able to identify more accurately those patients with benefit (in terms of overall survival) from anti-angiogenic agents (including sunitinib). In keeping with our findings, a cut-off of 10% of the sum of the longest tumour diameter shrinkage on the first follow-up CT scan was predictive of outcome, however, some challenges existed such as the lack of placebo-treated patients which did not allow them to explore whether the new cut-off was a predictive factor or not (Chen *et al*, 2014). The fact that similar findings have been shown in small series of gastrointestinal NETs treated with somatostatin analogues provide robustness to our findings (Luo, 2017). In this series, Luo *et al* included 33 patients with NETs treated with SSA; the authors identified that achieving a response of 10% reduction in target lesions impacted PFS.

Our study has some note-worthy strengths such as the fact that all data were prospectively collected as part of phase II and phase III clinical trials, in addition to the previous quality-assurance of these data for registration purposes. We also explored first which was the most informative time-point, in order to calculate the response cut-off at such time-point; this, was one of the acknowledged limitations of the previous studies in RCC (Chen *et al*, 2014). Although the use of morphological changes (such as Choi criteria (van der Veldt *et al*, 2010)) have been suggested in order to improve assessment of response to targeted therapies, the incorporation of such approaches to daily practice could be challenging due to the fact that it requires specialist radiological input for assessment of changes within density of target lesions (Faivre *et al*, 2012). We do therefore believe that the application of our 10% alternative cut-off could be relatively straight-forward for clinicians managing patients with pNETs in daily practice.

Limitations of our study include the fact that our analysis was limited to the measurement of marker lesions; appearance of new lesions could not be included in our analysis since it is not included in the calculation of change in percentages of response. Whether this radiological response is a predictive factor in addition to prognostic remains unclear, since patients treated with placebo who achieved such response did benefit in terms of PFS as well. It is worth highlighting the fact that the phase III study included in this analysis was interrupted early by the independent data monitoring committee (IDMC); and that following final results and demonstration of superiority of sunitinib, cross-over was allowed. Thus, the fact that some of the patients initially allocated to the placebo arm will have been treated with sunitinib (including patients in the absence of disease progression) could also explain why some patients in the placebo arm did have radiological response to treatment and its impact on prognosis regardless of the treatment group (as shown in the multivariable analysis). This would warrant further investigations in future placebo-controlled clinical trials. Another of the limitations from our study was that different sunitinib schedules were used across the phase II and the phase III studies. We do wonder whether this could be the explanation why we were unable to replicate the differences in median PFS found in the phase III study when comparing patients who did/did not reach the 10% alternative cut-off in the phase II patient population. The higher partial response rate identified in the phase II study patients could have also contributed to this. Finally, since the limited sample size did not allow us to divide our sample in separate design and validation cohorts, our results should be validated in future prospective series or clinical trials. Finally, it could also be argued that the 10% reduction in sum of marker lesions might be included within the expected inter-observer and inter-examination variability, especially when CT scans are performed as part of the daily practice and assessed outside the setting of a prospective clinical trial. It is worth highlighting that, although we do agreed with this being a possibility we do believe it is unlikely to happen due the fact that the CT scans employed in this study were not centrally reviewed and that the assessments and measurements were based on local radiologist (therefore reflecting standard clinical practice).

In conclusion, our results support that reduction of 10% in the measurement of marker lesions, impacts on PFS and should be considered enough to classify pNET patients as responders to sunitinib and likely to derive clinical benefit from treatment.

## Figures and Tables

**Table 1:** Baseline characteristics of the patients included in our analysis

	**All patients (*****n*****=237)**	**Sunitinib group (*****n*****=152)**	**Control group (Placebo;** ***n*****=85)**
**Patient demographics**			
Age at study entry (years)			
Median (range)	56 (25–84)	56 (25–84)	57 (26–78)
⩾ 65 years old	55 (23%)	32 (21%)	23 (27%)
Gender			
Male (*n* (%))	124 (52%)	84 (55%)	40 (47%)
Female (*n* (%))	113 (48%)	68 (45%)	45 (53%)
Race			
White (*n* (%))	160 (67%)	107 (70%)	53 (62%)
Black (*n* (%))	4 (2%)	4 (3%)	0 (0%)
Asian (*n* (%))	24 (10%)	14 (9%)	10 (12%)
Other (*n* (%))	49 (21%)	27 (18%)	22 (26%)
ECOG PS at study entry			
0 (*n* (%))	129 (54%)	88 (59%)	41 (48%)
1 (*n* (%))	107 (45%)	64 (42%)	43 (51%)
2 (*n* (%))	1 (1%)	0 (0%)	1 (1%)
Functioning tumour			
Yes (*n* (%))	104 (44%)	63 (41%)	41 (52%)
No (*n* (%))	132 (55%)	88 (58%)	44 (48%)
Unknown (*n* (%))	1 (1%)	1 (1%)	0 (0%)
First diagnosis ≥3 years before inclusion in trial			
Yes (*n* (%))	103 (43%)	55 (36%)	48 (56%)
No (*n* (%))	133 (56%)	96 (63%)	37 (44%)
Unknown (*n* (%))	1 (1%)	1 (1%)	0 (0%)
Previous systemic treatment (excluding SSA)			
Yes (*n* (%))	135 (57%)	85 (56%)	50 (59%)
No (*n* (%))	102 (43%)	67 (44%)	35 (41%)
Is sunitinib/placebo 3rd line of treatment or more (excluding SSA)?			
Yes (*n* (%))	70 (30%)	45 (30%)	25 (29%)
No (*n* (%))	167 (70%)	107 (70%)	60 (71%)
Dose of sunitinib/placebo			
37.5 mg od	171 (72%)	86 (57%)	85 (100%)
50 mg od (4+2)	66 (28%)	66 (43%)	0 (0%)
**Patient outcomes**			
Follow-up, median (range)	16.5 (0.1–80.6)	15.6 (0.1–80.3)	23.2 (0.1–80.6)
PFS (estimated median KM)^a^, median (95% CI)	7.7 (95%-CI 7.1–9.3)	9.3 (95% CI 7.6–12.2)	5.4 (3.5–6.01)
Events PFS, yes (*n* (%))	112 (47%)	63 (41%)	49 (58%)
Free-of progression at 11 months, yes (*n* (%))	32 (14%)	27 (18%)	5 (6%)
Median time on treatment (months), median (range)	5.1 (0–20.1)	6.4 (0.4–19.7)	3.7 (0–20.1)
Best radiological response (defined by local investigator)^a^			
Partial response (*n* (%))	19 (8%)	19 (13%)	0 (0%)
Stable disease (*n* (%))	150 (63%)	101 (66%)	49 (58%)
Progressive disease (*n* (%))	35 (15%)	14 (9%)	21 (25%)
Indeterminate (*n* (%))	33 (14%)	18 (12%)	15 (17%)
Best change in sum of marker lesions (%)^b^, median (range)	-5.7 (-100–43.3%)	-12.8 (-100–36.4)	1.7 (-46.7–43.3)
Time of scan with best-response^b^, median (range)	1 (1–17)	3 (1–17)	1 (1–13)
Number of scans available^b^, total number	788	535	253

Abbreviations: ECOG-PS=Eastern Cooperative Oncology Group performance status; KM=Kaplan–Meier estimation; n=number of patients; PFS=progression-free survival; SSA=somatostatin analogue.

a As defined by local investigator.

b As per calculations performed in this *post hoc* analysis.

**Table 2:** Identification of the most informative time-point employing ROC curve analysis (AUC) and logistic regression for prediction of progression-free at 11 months

		**ROC analysis**	**Logistic regression**		**ROC curve comparison analysis**	
**Time-point**	**Number of observations**	**AUC (95% CI)**	**Logistic Regression OR (**95% **CI)**	**Logistic Regression** ***P*****-value**	**AUC (95% CI)**	**Number of observation**
Best response	144	0.77 (0.67–0.86)	1.05 (1.02–1.07)	<0.001	0.66 (0.53–0.79)^a^	68
Month 1	134	0.67 (0.54–0.78)	1.05 (1.01–1.1)	0.011	Not included	—
Month 2	68	0.57 (0.43–0.72)	1.01 (0.98–1.04)	0.489	Not included	—
Month 3	107	0.60 (0.48–0.74)	1.02 (1.01–1.04)	0.037	Not included	—
Month 5	79	0.76 (0.65–0.86)	1.04 (1.02–1.06)	0.001	0.74 (0.62–0.86)^a^	68
Month 7	54	0.78 (0.66–0.9)	1.05 (1.01–1.08)	0.002	0.75 (0.63–0.86)^a^	68
Month 9	38	0.73 (0.54–0.91)	1.04 (0.99–1.1)	0.078	Not included	—
Month 11	24	0.64 (0.26–1)	1.02 (0.98–1.06)	0.370	Not included	—
Month 13	18	0.49 (0.1–0.96)	0.99 (0.97–1.02)	0.553	Not included	—
Month 15	8	0.60 (0–1)	0.98 (0.94–1.03)	0.496	Not included	—
Month 17	2	—	—	—	Not included	—
Month 19	2	—	—	—	Not included	—
Month 21	1	—	—	—	Not included	−

Abbreviations: AUC=area under the curve; CI=confidence interval; OR=odds ratio; ROC=receiver operating characteristic.

a Chi-square *P*-value 0.3733 (Supplementary Figure 3).

**Table 3:** Identification of alternative cut-offs for definition of ‘partial response’ employing data from the 54 scans available at month 7

**Cut-off**	**Sensitivity**	**Specificity**	**Correctly classified patients (%)**	**Number of patients classified as response when this cut-off is employed to the best-response assessment**^a^
−30%	31%	96%	64.8%	29 (20%)
−20%	39%	89%	64.8%	51 (35%)
−15%	46%	86%	66.7%	61 (42%)
−10%	50%	82%	66.7%	84 (59%)

a As per calculations performed in this *post hoc* analysis.

**Table 4:** Survival analyses: median PFS estimations (Kaplan–Meier method) and univariate and multivariable Cox regression analyses

**Univariate analysis (by type of treatment and response)**						
**Sunitinib-treated patients**	**Variable**	**Group**	**Median PFS in the responding group (months) (KM)**	**Median PFS in the non-responding group (months)**	**Univariate COX (HR** **95%** **CI)**	**Univariate COX** ***P*****-value**
All patients treated with Sunitinib (*n*=144)	Best-response cut-off	−30%	12.23 (8.32–14.99)	8.28 (7.10–12.49)	0.69 (0.38–1.22)	0.198
		−10%	10.98 (8.28–12.71)	7.63 (6.54–12.92)	0.55 (0.31–0.97)	0.040
Phase III patients treated with Sunitinib (*n*=80)	Best-response cut-off	−30%	13.60 (7.49-nr)	10.15 (7.19-nr)	0.68 (0.28–1.62)	0.386
		−10%	13.60 (7.39-nr)	6.01 (2.10-nr)	0.33 (0.15–0.72)	0.005
Phase II patients treated with Sunitinib (*n*=64)	Best-response cut-off	−30%	10.98 (7.79–14.99)	7.63 (6.61–12.26)	0.48 (0.21–1.13)	0.093
		−10%	9.30 (7.10–12.23)	7.63 (6.54-nr)	1.01 (0.42–2.43)	0.980
**Placebo-treated patients**	**Variable**	**Group**	**Median PFS in the responding group (months)**	**Median PFS in the non-responding group (months)**	**Univariate COX (HR 95%-CI)**	**Univariate COX** ***P*****-value**
Phase III patients treated with Placebo (*n*=76)	Best response cut-off	−30%	Nr (nr-nr)	5.42 (3.38–6.01)	0.59 (0.08–4.43)	0.616
		−10%	9.63 (9.63-nr)	3.84 (3.38–5.65)	0.30 (0.09–0.99)	0.050
**Univariate/multivariable anlaysis (by type of treatment and response)**						
**Variable**	**Group**	**Median PFS (months) KM**	**Univariate COX-regression**		**Multivariable COX-Regression (*****n*****=220)**	
			HR (95% CI)	*P*-value	HR (95%-CI)	*P*-value
Treatment	Placebo	5.42 (3.48–6.01)	1 (Ref.)	−	1 (Ref.)	−
	Sunitinib	9.30 (7.63–12.26)	0.43 (0.29–0.62)	<0.001	0.56 (0.36–0.89)	0.014
Classical cut-off (30%)	No	7.39 (6.01–8.27)	1 (Ref.)	−	1 (Ref.)	−
	Yes	11.97 (9.27–14.99)	0.56 (0.34–0.93)	0.026	0.95 (0.52–1.75)	0.877
Alternative Cut-off (10%)	No	5.85 (3.98–7.39)	1 (Ref.)	−	1 (Ref.)	−
	Yes	10.99 (8.32–12.2)	0.42 (0.28–0.62)	<0.001	0.57 (0.34–0.97)	0.038

Abbreviations: CI=confidence interval; ECOG PS=Eastern Cooperative Oncology Group Performance Status; HR=hazard ratio; KM=Kaplan–Meier estimation; n=number of patients; NR=not reached; PFS=progression-free survival; Ref.=reference category.
