# Supplementary figures and images for: Determination of an optimal response cut-off able to predict progression-free survival in patients with well-differentiated advanced pancreatic neuroendocrine tumours treated with sunitinib: an alternative to the current RECIST-defined response
Source: Br J Cancer. 2017 Nov 21;118(2):181–8. doi: 10.1038/bjc.2017.402 (PMC5785750; doi:10.1038/bjc.2017.402)

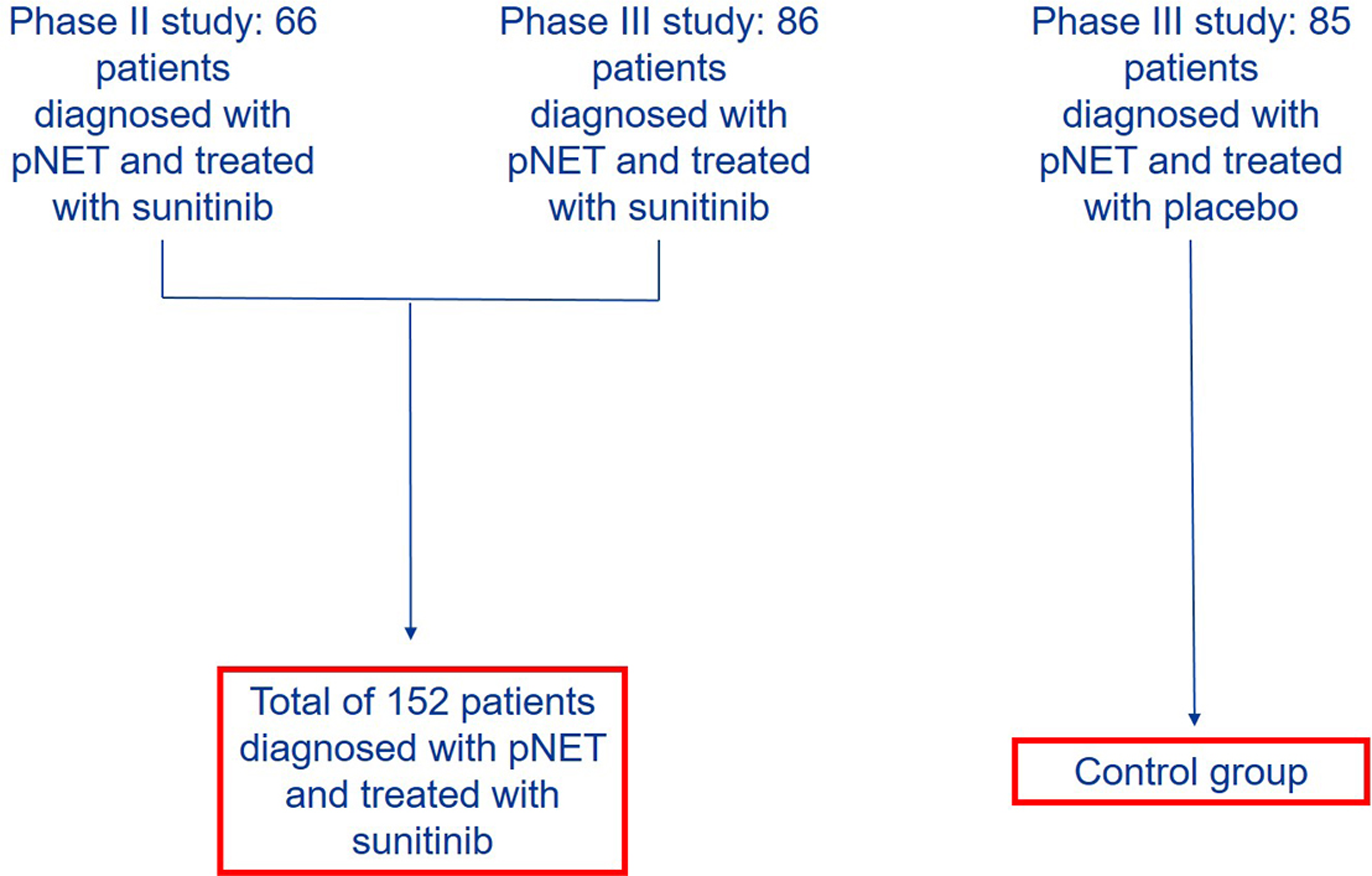

Supplement: Supplementary Figure 1 [file bjc2017402x1.tif]

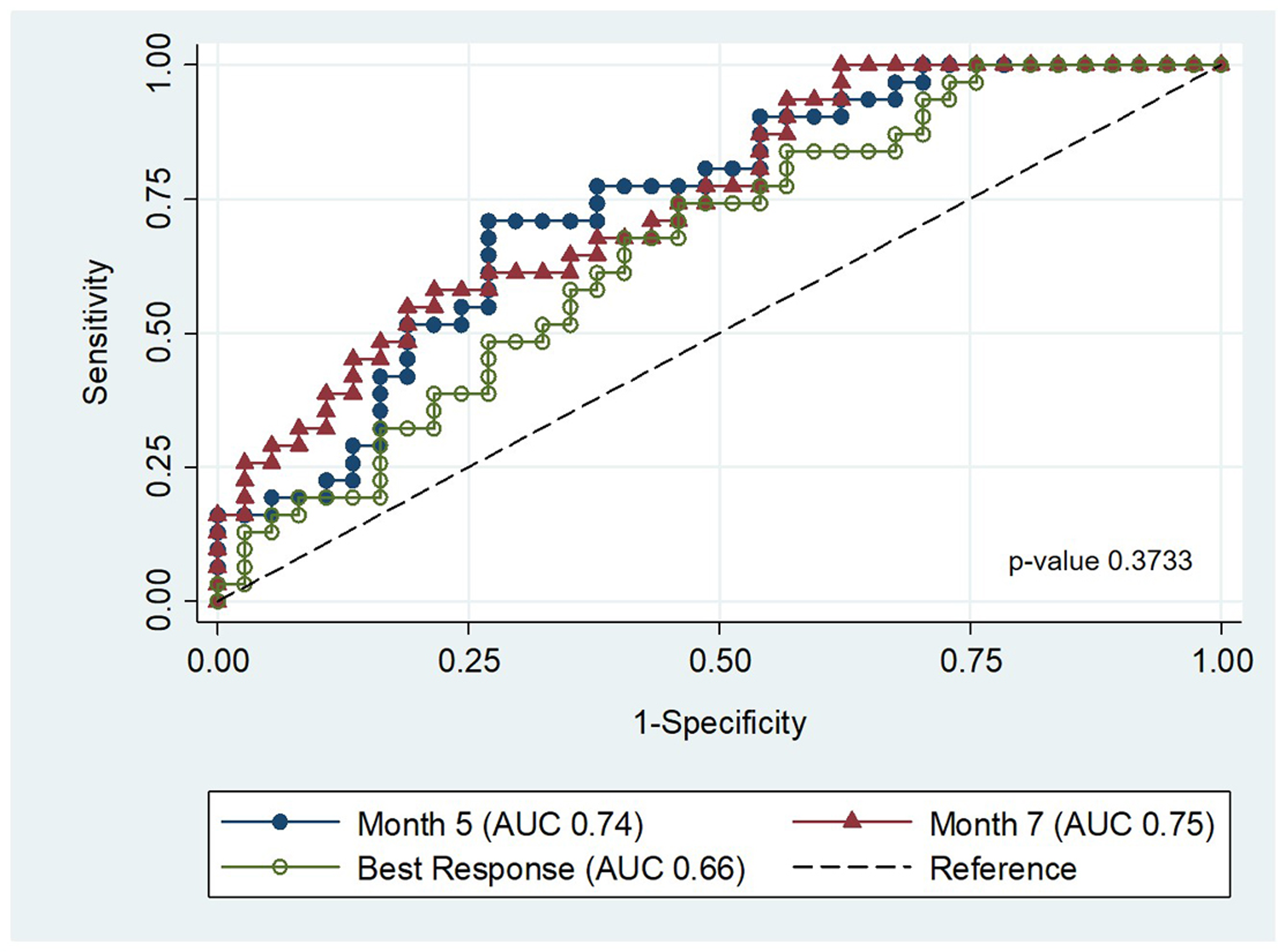

Supplement: Supplementary Figure 2 [file bjc2017402x2.tif]
