# Supplementary material for: Determination of an optimal response cut-off able to predict progression-free survival in patients with well-differentiated advanced pancreatic neuroendocrine tumours treated with sunitinib: an alternative to the current RECIST-defined response
Source: Br J Cancer. 2017 Nov 21;118(2):181–8. doi: 10.1038/bjc.2017.402 (PMC5785750; doi:10.1038/bjc.2017.402)
Supplement: Supplementary Table 1 [file bjc2017402x3.docx]

|  |  | **All patients (n=237)** | **Sunitinib group (n=152)** | **Sunitinib (Phase II) (n=66)** | **Sunitinib (Phase III) (n=86)** | **Control group (Placebo; n=85)** |
| --- | --- | --- | --- | --- | --- | --- |
| **Follow-up** | Median (range) | 16.5 (0.1-80.6) | 15.6 (0.1-80.3) | 12 (1.4-28.3) | 32.5 (0.7-80.3) | 23.2 (0.1-80.6) |
| **PFS (estimated median KM)^$^** | Median (95%-CI) | 7.7 (95%-CI 7.1-9.3) | 9.3 (95%-CI 7.6-12.2) | 9.3 (95%-CI 7.1-11.9) | 12.6 (95%-CI 7.4-16.8) | 5.4 (3.5-6.01) |
| **Events PFS** | Yes (n (%)) | 112 (47%) | 63 (41%) | 33 (50%) | 30 (35%) | 49 (58%) |
| **Free-of progression at 11 months** | Yes (n (%)) | 32 (14%) | 27 (18%) | 10 (15%) | 17 (20%) | 5 (6%) |
| **OS (estimated median KM)^$^** | Median (95%-CI) | 20.6 (95%-CI 16.6-26.7) | 19.3 (95%-CI 15.5-23.5) | 12.5 (95%-CI 11.4-13.5) | 38.6 (95%-CI 21.5-56.01) | 29.1 (95%-CI 16.4-36.5) |
| **Partial response (RECIST v1.0) (defined by local investigator)^$^** | Yes (n (%)) | 19 (8%) | 19 (13%) | 11 (17%) | 8 (9%) | 0 (0%) |
| **Median reduction (%)*** | Median (range) | -5.7 (-100-43.3%) | -12.8 (-100-36.4) | -13.1 (-76-21.1) | -12.4 (-100-36.4) | 1.7 (-46.7-43.3) |
