# Supplementary material for: Determination of an optimal response cut-off able to predict progression-free survival in patients with well-differentiated advanced pancreatic neuroendocrine tumours treated with sunitinib: an alternative to the current RECIST-defined response
Source: Br J Cancer. 2017 Nov 21;118(2):181–8. doi: 10.1038/bjc.2017.402 (PMC5785750; doi:10.1038/bjc.2017.402)
Supplement: Supplementary Table 2 [file bjc2017402x4.docx]

| **Time-point** | **All patients** | | **Patients with Partial Response** | | **Patients with Stable Disease** | | **Patients with Progressive disease** | |
| --- | --- | --- | --- | --- | --- | --- | --- | --- |
|  | N | % | N | % | N | % | N | % |
| **TOTAL of patients** | 237 | 100 | 19 | 100 | 150 | 100 | 35 | 100 |
| **Month 1** | 99 | 41.77 | 0 | 0 | 56 | 37.33 | 29 | 82.86 |
| **Month 2** | 28 | 11.81 | 1 | 5.26 | 21 | 14 | 4 | 11.43 |
| **Month 3** | 44 | 18.57 | 8 | 42.11 | 34 | 22.67 | 2 | 5.71 |
| **Month 5** | 18 | 7.59 | 3 | 15.79 | 15 | 10 | 0 | 0 |
| **Month 7** | 10 | 4.22 | 1 | 5.26 | 9 | 6 | 0 | 0 |
| **Month 9** | 8 | 3.38 | 3 | 15.79 | 5 | 3.33 | 0 | 0 |
| **Month 11** | 3 | 1.27 | 1 | 5.26 | 2 | 1.33 | 0 | 0 |
| **Month 13** | 6 | 2.53 | 2 | 10.53 | 4 | 2.67 | 0 | 0 |
| **Month 15** | 2 | 0.84 | 0 | 0 | 2 | 1.33 | 0 | 0 |
| **Month 17** | 1 | 0.42 | 0 | 0 | 1 | 0.67 | 0 | 0 |
| **Missing** | 18 | 7.59 | 0 | 0 | 1 | 0.67 | 0 | 0 |
| **Month 7 or earlier** | 199 | 83.96 | 13 | 68.42 | 135 | 90 | 35 | 100 |
