# Supplementary material for: Determination of an optimal response cut-off able to predict progression-free survival in patients with well-differentiated advanced pancreatic neuroendocrine tumours treated with sunitinib: an alternative to the current RECIST-defined response
Source: Br J Cancer. 2017 Nov 21;118(2):181–8. doi: 10.1038/bjc.2017.402 (PMC5785750; doi:10.1038/bjc.2017.402)
Supplement: Supplementary Table 3 [file bjc2017402x5.docx]

| **Month 5** | **Cut-off** | **Sensitivity** | **Specificity** | **Correctly classified patients (%)** |
| --- | --- | --- | --- | --- |
|  | **-30%** | 33% | 84% | 67.1% |
|  | **-20%** | 44% | 77% | 65.8% |
|  | **-15%** | 52% | 75% | 67.1% |
|  | **-10%** | 63% | 69% | 67.1% |
