# Supplementary material for: Determination of an optimal response cut-off able to predict progression-free survival in patients with well-differentiated advanced pancreatic neuroendocrine tumours treated with sunitinib: an alternative to the current RECIST-defined response
Source: Br J Cancer. 2017 Nov 21;118(2):181–8. doi: 10.1038/bjc.2017.402 (PMC5785750; doi:10.1038/bjc.2017.402)
Supplement: Supplementary Table 4 [file bjc2017402x6.docx]

| **Variable** | **Group** | **Median PFS (months) KM** | **Univariate COX-regression** | | **Multivariable COX-Regression (n=220)** | |
| --- | --- | --- | --- | --- | --- | --- |
|  |  |  | HR (95%-CI) | p-value | HR (95%-CI) | p-value |
| **Treatment** | **Placebo** | 5.42 (3.48-6.01) | 1 (Ref) | - | 1 (Ref) | - |
|  | **Sunitinib** | 9.3 (7.63-12.26) | 0.43 (0.29-0.62) | <0.001 | 0.56 (0.36-0.89) | 0.014 |
| **Classical Cut-off (30%)** | **No** | 7.39 (6.01-8.27) | 1 (Ref) | - | 1 (Ref) | - |
|  | **Yes** | 11.97 (9.27-14.99) | 0.56 (0.34-0.93) | 0.026 | 0.95 (0.52-1.75) | 0.877 |
| **Alternative Cut-off (10%)** | **No** | 5.85 (3.98-7.39) | 1 (Ref) | - | 1 (Ref) | - |
|  | **Yes** | 10.99 (8.32-12.2) | 0.42 (0.28-0.62) | <0.001 | 0.57 (0.34-0.97) | 0.038 |
| **Age at study entry (years)** | **Continuous variable** | n/a | 0.98 (0.96-1.01) | 0.058 | - | - |
| **Gender** | **Male** | 7.49 (6.54-8.38) | 1 (Ref) | - | - | - |
|  | **Female** | 10.98 (6.61-12.49) | 0.89 (0.61-0.30) | 0.553 | - | - |
| **Race** | **White** | 8.38 (7.39-11.07) | 1 (Ref) | - | - | - |
|  | **Black** | 6.61 (3.72-nr) | 1.43 (0.45-4.58) | 0.542 | - | - |
|  | **Asian** | 8.32 (1.81-nr) | 0.64 (0.32-1.29) | 0.213 | - | - |
|  | **Other** | 6.01 (3.75-7.49) | 1.32 (0.86-2.02) | 0.211 | - | - |
| **ECOG PS at study entry** | **0** | 7.63 (7.10-10.15) | 1 (Ref) | - | - | - |
|  | **≥1** | 7.92 (6.01-11.37) | 1.01 (0.69-1.45) | 0.957 | - | - |
| **Functioning tumour** | **No** | 7.66 (6.61-9.63) | 1 (Ref) | - | - | - |
|  | **Yes** | 7.49 (5.85-11.59) | - 1. (0.68-1.49) | 0.987 | - | - |
| **First diagnosis ≥3 years before inclusion in trial** | **No** | 7.39 (6.51-7.92) | 1 (Ref) | - | - | - |
|  | **Yes** | 9.63 (7.19-11.96) | 0.76 (0.52-1.11) | 0.154 | - | - |
| **Previous systemic treatment (excluding SSA)** | **No** | 9.27 (7.10-11.37) | 1 (Ref) | - | - | - |
|  | **Yes** | 7.42 (6.54-9.30) | 1.01 (0.68-1.48) | 0.977 | - | - |
| **Is sunitinib/placebo 3^rd^ line of treatment or more (excluding SSA)?** | **No** | 7.67 (6.74-10.15) | 1 (Ref) | - | - | - |
|  | **Yes** | 7.39 (6.01-10.98) | 0.93 (0.61-1.41) | 0.719 | - | - |
