# Supplementary material for: Determination of an optimal response cut-off able to predict progression-free survival in patients with well-differentiated advanced pancreatic neuroendocrine tumours treated with sunitinib: an alternative to the current RECIST-defined response
Source: Br J Cancer. 2017 Nov 21;118(2):181–8. doi: 10.1038/bjc.2017.402 (PMC5785750; doi:10.1038/bjc.2017.402)
Supplement: Supplementary Information Legends [file bjc2017402x7.docx]

Supplementary Figure 1 – Data for 237 patients was received

Supplementary Figure 2 – Receiver operating characteristic (ROC) curve comparison analysis

Supplementary Table 1 – Differences in patients’ outcomes between sunitinib-treated patients in phase II and phase III studies

Supplementary Table 2 – Time-point when best response (defined by local investigator) was achieved

Supplementary Table 3 – Performance of alternative cut-off in month 5 (79 scans) was tested

Supplementary Table 4 – Univariate and multivariable Cox regression analyses (all patients; all variables)
